# Increasing Embryonic Morphogen Nodal Expression Suggests Malignant Transformation in Colorectal Lesions and as a Potential Marker for CMS4 Subtype of Colorectal Cancer

**DOI:** 10.3389/pore.2021.587029

**Published:** 2021-03-10

**Authors:** Xiaopai Wang, Shousheng Liu, Huijiao Cao, Xiubo Li, Yuming Rong, Guorong Liu, Hong Du, Hong Shen

**Affiliations:** ^1^ Department of Pathology, School of Basic Medical Sciences, Southern Medical University, Guangzhou, China; ^2^ Department of Pathology, Guangzhou First People's Hospital, School of Medicine, South China University of Technology, Guangzhou, China; ^3^ Department of General Medicine, State Key Laboratory of Oncology in South China, Collaborative Innovation Center for Cancer Medicine, Sun Yat-sen University Cancer Center, Guangzhou, China

**Keywords:** Nodal, TGF-β, colorectal cancer, CMS-4, tumor-stroma percentage

## Abstract

Nodal, an embryonic morphogen in TGF-β family, is related with tumorigenicity and progression in various tumors including colorectal cancer (CRC). However, the difference of Nodal expression between CRC and colorectal polyps has not yet been investigated. Besides, whether Nodal can be used as a marker for consensus molecular subtype classification-4 (CMS4) of CRC is also worth studying. We analyzed Nodal expression in patients of CRC (161), high-grade intraepithelial neoplasia (HGIN, 28) and five types of colorectal polyps (116). The Nodal expression difference among groups and the association between Nodal expression and clinicopathological features were analyzed. Two categories logistic regression model was used to predict the odds ratio (OR) of risk factors for high tumor-stroma percentage (TSP), and ROC curve was used to assess the diagnostic value of Nodal in predicting high TSP in CRC. We found that Nodal expression was significantly elevated in CRC and HGIN (*p* < 0.0001). The increased expression of Nodal was related with high TSP, mismatch repair-proficient (pMMR) status, lymph node metastasis and advanced AJCC stage (*p* < 0.05). Besides, Nodal expression was the only risk factor for high TSP (OR = 6.94; *p* < 0.001), and ROC curve demonstrated that Nodal expression was able to efficiently distinguish high and low TSP. In conclusion, different expression of Nodal between CRC/HGIN and benign lesions is suggestive of a promoting role for Nodal in colorectal tumor progression. Besides, Nodal might also be used as a potential marker for CMS4 subtype of CRC.

## Introduction

As is well-known, colorectal cancer (CRC) is a heterogeneous disease with complicated molecular profile, and the overall survival of CRC patients in advanced stage remains poor, with an approximate 50% overall 5-years survival rate [1]. Vogelstein et al recorded the model of gradual step-wise accumulation of epigenetic and genetic events leading to adenoma and carcinoma occurrence, and also put forward a new review about the function of ‘driver’ alterations in tumor suppressor genes such as *SMAD4* and *APC*, and oncogenes such as *PIK3CA*, *KRAS* and *BRAF* that screen for advantageous genes and result in CRC progression [1, 2]. More and more researches focus on the molecular changes in CRC tumorigenesis and progression in order to identify novel diagnostic and therapeutic targets for CRC.

The human Nodal gene located on chromosome 10q22, is a member of the Transforming Growth Factor Beta (TGF-β) superfamily and plays a critical role in maintaining pluripotency of human embryonic stem cells (hESCs) and mesodermal differentiation, including epithelial-to-mesenchymal transition (EMT) [3]. Normally, Nodal expression is largely limited to embryonic tissues and not usually expressed in adult tissues [4]. Recently, more and more findings have revealed that Nodal reemerged in a number of tumors such as CRC, melanoma, breast cancer and prostate cancer [5–8]. The initial researches have revealed that Nodal was higher in human colon cancer tissues than that in adjacent noncancerous colon tissues, and Nodal was shown to accelerate self-renewal of human colon cancer stem cells via Smad2/3 signaling pathway [8]. In addition, Nodal and Aldehyde Dehydragenase-1 can served as prognostic markers for CRC [9]. However, the Nodal expression in colorectal polyps remains unclear. Therefore, we inspected and compared Nodal expression in a wide spectrum of intestinal polyps and CRC using immunohistochemistry (IHC) method.

In 2015, the consensus molecular subtype (CMS) classification of CRC was reported [10], which represented the best description of CRC heterogeneity and might be applied to guide the target therapy in the future. There have been established four CMSs: CMS1 (MSI Immune, 14%) is characterized as exhibiting microsatellite instability (MSI), immune activation and CpG island methylation phenotype (CIMP); CMS2 (Canonical, 37%) is characterized as showing somatic copy number analysis (SCNA), WNT/MYC signaling pathway activation and microsatellite stable (MSS) status; CMS3 (Metabolic, 13%) is characterized as exhibiting evident metabolic dysregulation and *KRAS* and *APC* mutations; and CMS4 (Mesenchymal, 23%) is characterized as showing TGF-β activation, stromal invasion and angiogenesis [11]. Particularly, CMS4 usually occurs at advanced stage with poorer prognosis than the other subtypes [12, 13]. The recently reported tumor-stroma percentage (TSP) was used to evaluate the proportions of tumor area infiltrated by stroma, and high TSP (>50%) is an appropriate marker to determine CMS4 subtype in the clinicopathologic diagnosing work [14]. Nevertheless, when tumor specimens contain more necrosis or mucus tissue, grading TSP is very difficult and it is meaningful to seek a new marker for assessing CMS4 subtype. As a member of TGF-β superfamily, whether Nodal expression has any relationship with TSP and thus can be used as a marker for CMS4 subtype? The present study also aimed to answer this question.

## Materials and Methods

### Patients and Specimens

Tissue samples were obtained from the department of pathology of Guangzhou First People’s hospital. A total of 305 lesions were collected in this study between June 2017 and March 2018, including 18 juvenile polyps (JPs), 22 hyperplastic polyps (HPs), 22 inflammatory polyps (IPs), 24 sessile serrated adenoma/polyps (SSA/Ps), 30 tubular adenomas (TAs), 28 high-grade intraepithelial neoplasms (HGINs), 143 primary CRCs and 18 metastatic CRCs (pulmonary and liver metastases). Besides, a total number of 20 normal colon tissues were collected and used as control. All the CRC patients had undergone surgical operation and none of them had received radiotherapy or chemotherapy before surgery. Tumor type and grade were evaluated according to the 2016 World Health Organization (WHO) classification system. The pathological tumor stage was defined according to the seventh edition of the tumor-node-metastasis (TNM) staging of the American Joint Cancer Committee (AJCC). Primary colon cancer was classified into left-sided (including descending colon and sigmoid) and right-side (including caecum, ascending and transverse colon) tumors.

### Immunohistochemistry Staining

Formalin-fixed and paraffin-embedded samples were cut into 4-μm thickness, deparaffinized in xylene, washed and dehydrated with graded ethanol, followed by antigen retrieval using high-pressure method with EDTA 9.0. The slides were then pretreated with 3% H_2_O_2_ for 10 min to block endogenous peroxidase activity and washed by PBS for three times. Afterward, the tissues were incubated with mouse monoclonal anti-Nodal antibody (1:300 dilution; ab55676; Abcam) at 37°C in water bath kettle for 45 min, following a 20 min incubation with biotin-linked secondary antibody (1:1000 dilution; ab47844; Abcam) at 37°C according to the manufacturer’s instructions. Then the sections were stained in DAB (diaminobenzidine tetrahydrochloride, Dako) solution and counterstained with hematoxylin for 1 min. Slides were then washed and dehydrated in graded ethanol, ultimately mounted under a cover slip. Each slide was observed under a light microscope (magnification, ×100) by two pathologists to obtain coincident results.

Cytoplasmic staining was considered Nodal-positive. The immunostaining of Nodal was scored according to positive cell rate and staining intensity. Two senior pathologists (XPW and HD) independently recorded the IHC results. The staining intensity was graded as follows: 0 score, no staining; 1 score, weak staining with light brown; 2 scoresintermediate staining; 3 scores, strongly stained with dark brown. The percentage of positive cells was divided into the following levels: 0 point for no cells positive; 1 point for <10% cells positive; 2 points for 10–50%, and 3 points for >50% cells positive, respectively. The staining index (SI) was calculated as (score for staining intensity) × (score for percentage of positive cells) [15, 16]. SI was divided into low group (total score ≤ 4.5) and high group (total score > 4.5) for further analysis.

### Histopathological Scoring for TSP

The evaluation of TSP was conducted using microscopic analysis of 4-µm hematoxylin and eosin (H&E) stained sections from the most invasive part of CRC as previously described [17, 18]. All the tissues were fixed, dehydrated, paraffin-embedded and sectioned, followed by hematoxylin staining, washing under running water, hydrochloric acid alcohol differentiation, dehydration using graded ethanol and vitrification by dimethylbenzene. Then, the slides were covered with a glass slip. Using a × 5 objective, the invasive area with the desmoplastic stroma was selected. Subsequently, the fields where the stroma was infiltrated with small tumor nest were calculated using a × 10 objective, meanwhile ensuring that tumor cells were present at all four sides of the image (north–east–south–west) and excluding areas of necrosis or mucin. Two pathologists (XPW and HD) estimated all the samples respectively. The TSP value was divided into “stroma-high” (>50%) and “stroma-low” (≤50%) groups with a 50% cut-off value [14].

### Statistical Analysis

Statistical analysis was carried out using SPSS 22.0 software (SPSS Inc, United States). The difference of Nodal expression among various types of colorectal lesions was compared using the Mann-Whitney U test. The Pearson Chi-square test was used for assessing the correlation between Nodal expression and clinicopathological parameters. Two categories logistic regression model was used for univariate and multivariate analysis to predict the odds ratio (OR) of individual factors for high TSP. Only variables with *p* value of less than 0.1 in the univariate model were included for further analysis in the multivariate logistic model. The ROC curve was plotted to evaluate the diagnostic value of Nodal in predicting high TSP. A *p* value of less than 0.05 was considered statistically significant.

## Results

### Nodal Expression in Various Types of Colorectal Lesions and TSP in CRC Tissues

**FIGURE 1:**
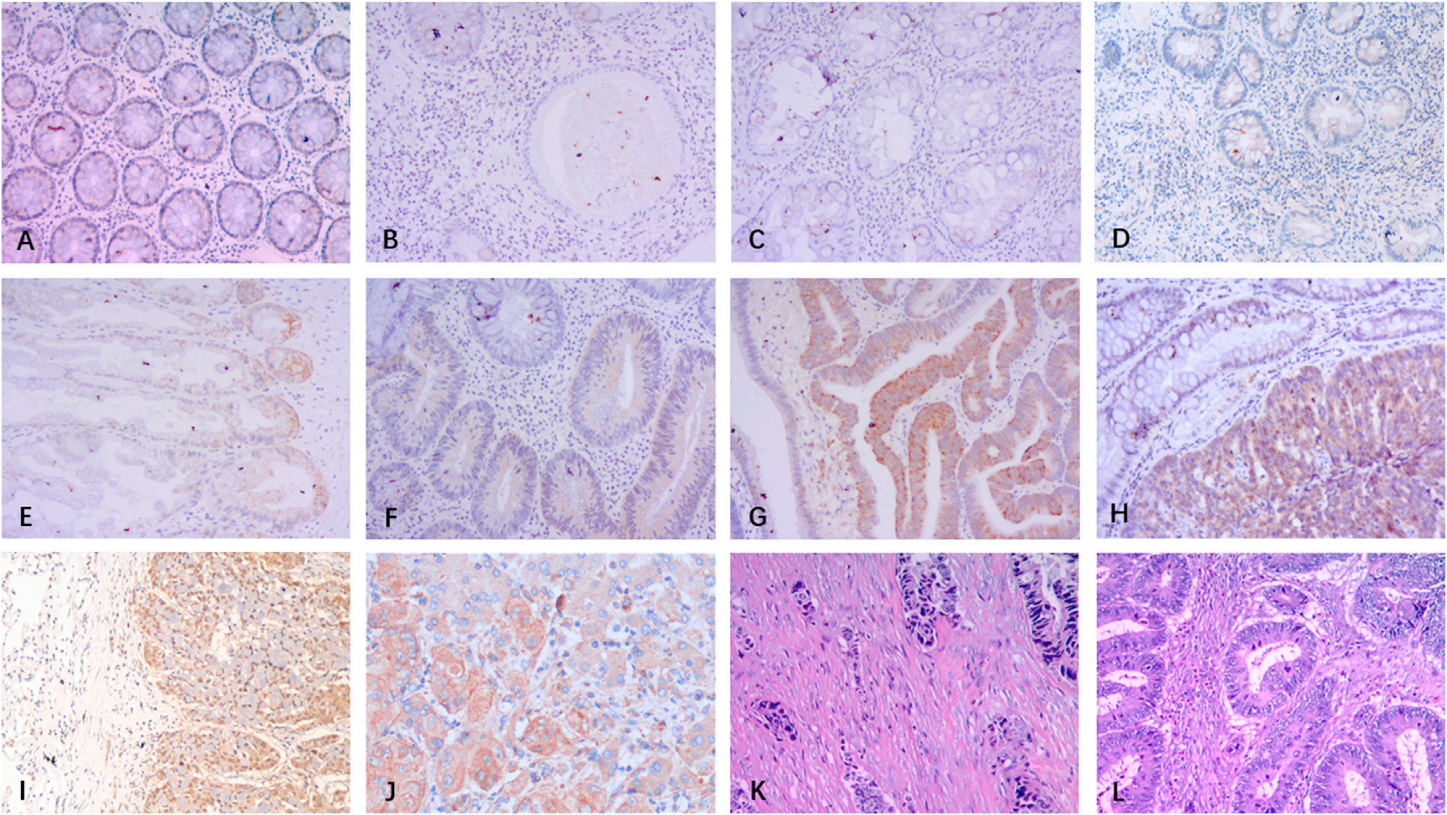
Nodal expression in normal colon tissue and various types of colorectal lesions and representative TSP in CRC. Nodal is hardly expressed in normal colon tissue **(A)**, juvenile polyp **(B)**, hyperplastic polyp **(C)** and inflammatory polyp **(D)**. Nodal expression is at low to moderate levels in sessile serrated adenoma/polyp **(E)** and tubular adenoma **(F)**. Nodal is significantly overexpressed in high-grade intraepithelial neoplasia **(G)**, primary tumor of CRC **(H)**, lung metastasis **(I)**, and liver metastasis **(J)** of CRC. CRC tumor stroma at invasive margin with high TSP (80%) and low TSP (20%) using HE staining **(K-L)** (100×).

Results of Nodal expression in various types of colorectal lesions are summarized in Table 1. Nodal was expressed at low levels in normal colon tissue (SI = 0.75 ± 0.38), JP (SI = 0.89 ± 0.42), HP (SI = 0.86 ± 0.37), and IP (SI = 0.75 ± 0.31) (Figures 1A–D), and there were no statistic differences among the four groups (all *p* > 0.05). SSA/P (SI = 2.17 ± 1.09) (Figure 1E) showed a weak to moderate Nodal expression which was comparable with TA (SI = 2.40 ± 1.22) (Figure 1F) whereas higher than the former four types (all *p* < 0.0001). Nodal expression was significantly increased in HGIN (SI = 4.18 ± 1.81), primary tumor of CRC (SI = 4.97 ± 2.27) as well as metastases of CRC (SI = 4.61 ± 2.55) (Figures 1G–J) compared with other lesions (all *p* < 0.0001), however, no significant differences were found between the three groups. Representative high (>50%) and low (≤50%) TSP in CRC tissues were displayed in Figures 1K,L).

**TABLE 1:** Statistical analysis of Nodal expression (staining index) using the Mann–Whitney *U*-test (*p*-value).

Group	NC (N = 20; SI = 0.75 ± 0.38)	JP (N = 18; SI = 0.89 ± 0.42)	HP (N = 22; SI = 0.86 ± 037)	IP (N = 22; SI = 0.75 ± 0.31)	SSA/P (N = 24; SI = 2.17 ± 1.09)	TA (N = 30; SI = 2.40 ± 1.22)	HGIN (N = 28; SI = 4.18 ± 1.81)	CRC (N = 143; SI = 4.97 ± 2.27)	mCRC (N = 18; SI = 4.61 ± 2.55)
NC		0.066	0.079	0.977	<0.0001	<0.0001	<0.0001	<0.0001	<0.0001
JP	0.066		0.907	0.062	<0.0001	<0.0001	<0.0001	<0.0001	<0.0001
HP	0.079	0.907		0.074	<0.0001	<0.0001	<0.0001	<0.0001	<0.0001
IP	0.977	0.062	0.074		<0.0001	<0.0001	<0.0001	<0.0001	<0.0001
SSA/P	<0.0001	<0.0001	<0.0001	<0.0001		0.809	<0.0001	<0.0001	<0.0001
TA	<0.0001	<0.0001	<0.0001	<0.0001	0.809		<0.0001	<0.0001	<0.0001
HGIN	<0.0001	<0.0001	<0.0001	<0.0001	<0.0001	<0.0001		0.123	0.853
CRC	<0.0001	<0.0001	<0.0001	<0.0001	<0.0001	<0.0001	0.123		0.455
mCRC	<0.0001	<0.0001	<0.0001	<0.0001	<0.0001	<0.0001	0.853	0.455	

SI, staining index, SI values are expressed as the mean ± standard deviation; NC, normal colon tissue; JP, juvenile polyp; HP, hyperplastic polyp; IP, inflammatory polyp; SSA/P, sessile serrated adenoma/polyp; TA, tubular adenoma; HGIN, high-grade intraepithelial neoplasia; CRC, primary tumor of colorectal cancer; mCRC, metastases of colorectal cancer.

### Relationship between Nodal Expression and Clinicopathologic Features in CRC Patients

We then investigated whether Nodal expression had relationship with other clinical features in CRC patients. In the CRC cells, Nodal was predominantly located in the cytoplasm and evaluated by SI criteria. As shown in Table 2, Nodal expression was significantly correlated with primary tumor site, AJCC stage, node stage, MMR status and TSP (all *p* < 0.05). Higher Nodal expression was prone to be found in left-sided colon cancer (compared with right-sided colon and rectum cancer, *p* = 0.044), advanced AJCC stage (stage III + IV compared with stage I + II, *p* = 0.001), lymphatic metastasis (positive compared with negative, *p* = 0.011), pMMR status (compared with mismatch repair-deficient (dMMR) status, *p* = 0.025) and high TSP (compared with low TSP, *p* < 0.001).There were 29 CRC cases (29/143, 20.3%) with high TSP (>50%), among which only five cases showed low expression of Nodal (5/29, 17.2%).

**TABLE 2:** Clinicopathological characteristics of CRC patients based on Nodal expression.

Characteristics	Nodal expression		r	*p*-value
Number of cases (n, %)	Low (≤4.5)	High (>4.5)		
Age at diagnosis (years)				0.156
≤61	42 (56.0)	30 (44.1)		
>61	33 (44.0)	38 (55.9)		
Gender				0.337
Male	24 (32.0)	27 (39.7)		
Female	51 (68.0)	41 (60.3)		
Primary tumor site			0.166	**0.044**
Left colon	20 (26.7)	29 (42.6)		
Right colon + Rectum	55 (73.3)	39 (57.4)		
Stage (7th AJCC)			0.264	**0.001**
I + II	47 (62.7)	24 (35.3)		
III + IV	28 (37.3)	44 (64.7)		
Tumor stage				0.853
T1 + T2	13 (17.3)	11 (16.2)		
T3 + T4	62 (82.7)	57 (83.8)		
Node stage			0.207	**0.011**
Negative	51 (68.0)	32 (47.1)		
Positive	24 (32.0)	36 (52.9)		
Tumor histological grade				0.09
Well + Moderately	61 (81.3)	62 (91.2)		
Poorly + Mucinous	14 (18.7)	6 (8.8)		
*KRAS* mutation				0.857
No	43 (57.3)	40 (58.8)		
Yes	32 (42.7)	28 (41.2)		
*BRAF* mutation				0.189
No	69 (92.0)	66 (97.1)		
Yes	6 (8.0)	2 (2.9)		
MMR status			0.184	**0.025**
pMMR	55 (73.3)	60 (88.2)		
dMMR	20 (26.7)	8 (11.8)		
TSP			0.335	<**0.001**
≤50%	70 (93.3)	44 (64.7)		
>50%	5 (6.7)	24 (35.3)		

r, contingency coefficient. The bold type indicates that the P value is statistically significant.

### Risk Factor Analysis for High TSP

As Nodal expression significantly correlated with high TSP, we next conducted the univariate and multivariate logistic regression analysis to exploit the risk factors for high TSP. As shown in Table 3, pMMR status and high Nodal expression were two risk factors for high TSP in univariate model (all *p* < 0.1). However, when it came into the multivariate logistic regression model, high Nodal expression was the only significant risk factor for high TSP (OR = 6.94, 95% confidence interval (CI) = 2.45–19.69, *p* < 0.001).

**TABLE 3:** Logistic regression analysis of risk factors for high TSP.

Factors	Univariate analysis		Multivariate analysis	
	OR (95% CI)	*p*-value	OR (95% CI)	*p*-value
Age at diagnosis (>61)	1.57 (0.69–3.59)	0.281		
Gender (female)	0.89 (0.38–2.06)	0.775		
Primary tumor site (left colon)	1.47 (0.64–3.39)	0.368		
Stage (7th AJCC) (III + IV)	1.52 (0.67–3.47)	0.320		
Tumor stage (T3 + T4)	3.23 (0.71–14.61)	0.128		
Node stage (positive)	1.64 (0.72–3.73)	0.235		
Tumor histological grade (poorly + mucinous)	0.98 (0.30–3.19)	0.973		
*KRAS* mutation (yes)	0.67 (0.29–1.58)	0.363		
*BRAF* mutation (yes)	1.33 (0.26–6.98)	0.733		
MMR status (dMMR)	0.25 (0.06–1.13)	0.071	0.35 (0.07–1.68)	0.191
Nodal expression (>4.5)	7.64 (2.71–21.5)	<0.001	6.94 (2.45–19.69)	<**0.001**

OR, odds ratio; 95% CI, 95% Confidence interval.

Only the meaningful factors (*p* < 0.1) in univariate analysis were brought into the multivariate analysis.

### The Diagnostic Value of Nodal Expression in Predicting High TSP

**FIGURE 2:**
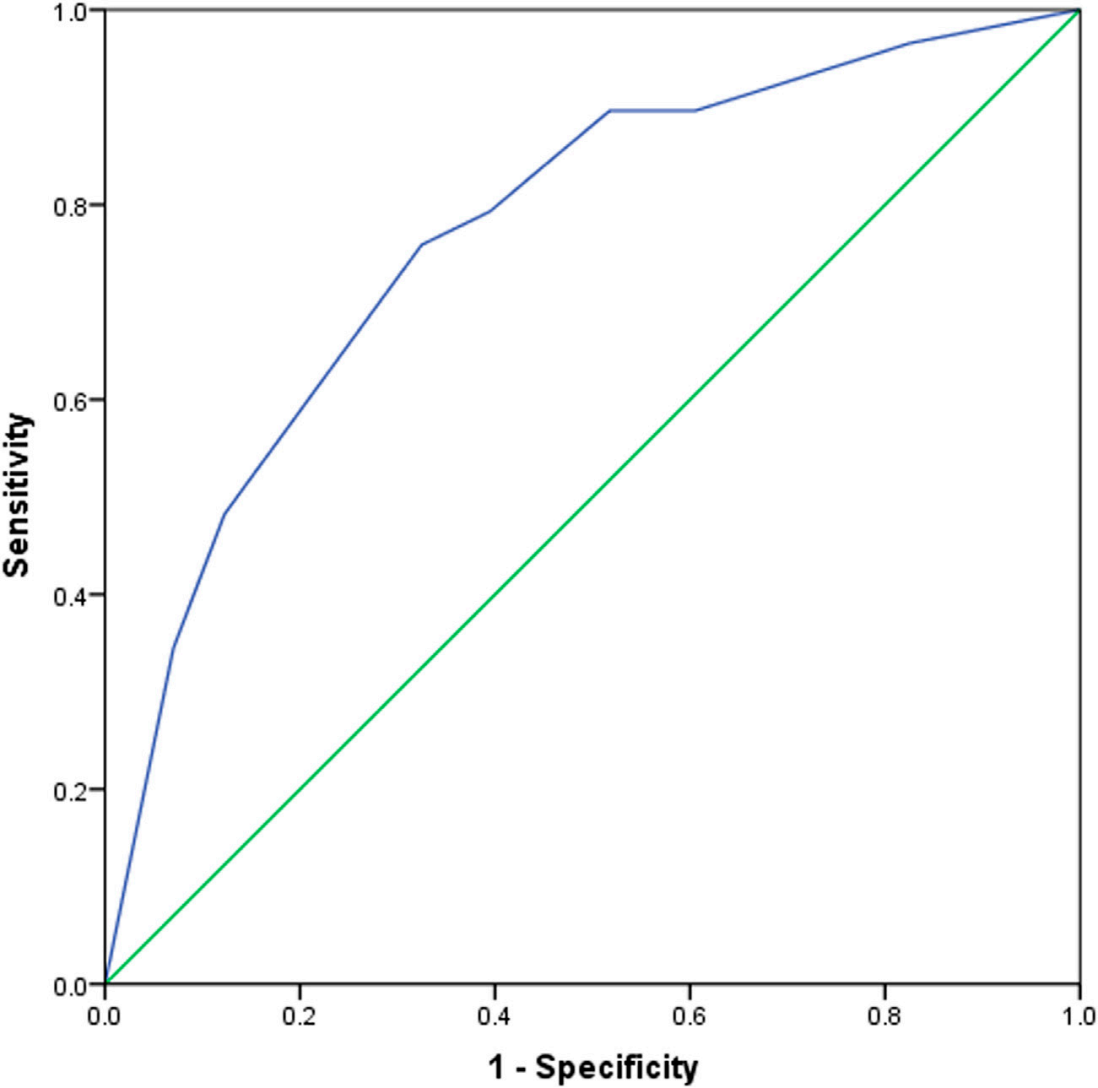
The diagnostic value of Nodal in patients with CMS4 CRC was analyzed using a ROC curve. The cutoff point was 5.50 and the area under the ROC curve (AUC) was 0.773 (*p* < 0.001, 95%CI = 0.676–0.869).

As high Nodal expression was the only significant risk factor for high TSP and high TSP is thought to be a good marker for determining CMS4 subtype of CRC in the routine pathology., we then plotted the ROC curve to assess the diagnostic value of Nodal in predicting high TSP or determining CMS4 subtype of CRC. As shown in Figure 2, the outcome demonstrated that Nodal expression was able to efficiently distinguish high and low TSP (AUC = 0.773, 95% CI = 0.676–0.869, *p* < 0.001). The optimal cut-off point of Nodal expression was 5.50, at which point a sensitivity of 75.9% and a specificity of 67.5% were obtained.

## Discussion

Nodal is a member of the TGF-β superfamily, as effective to both embryological development and tumorigenesis. Growing researches have reported that Nodal is expressed in various tumors. Nodal plays an important role in angiogenesis, invasion and progression of cancer, as upregulation of Nodal caused a loss of E-cadherin and upregulated mesenchymal markers including N-cadherin, Twist1 and Vimentin, inducing EMT via the ERK pathway, thus played a promoting role [19], while suppression of Nodal expression reduced the clonogenicity, tumorigenesis and metastasis abilities of cancer cells [20, 21].

In the present study, we found that Nodal expression prominently increased in HGIN and CRC, whereas was at lower level in SSA/P and TA, and hardly expressed in normal colon tissue, JP, HP and IP. Previous research about Nodal expression in cutaneous melanocytic lesions showed that Nodal expression was significantly increased in malignant lesions including malignant melanoma, metastatic melanoma and melanoma *in situ* compared with those benign melanocytic lesions (no expression or low expression), indicating a role for Nodal in melanoma progression [15]. Nodal expression also increased in triple-negative breast cancer biopsies, whereas it was hardly detectable in benign breast lesions [22]. Based on our research and previously reported studies, we speculate that increased Nodal expression might also play an important role in tumorigenesis and progression of CRC, whereas needs further research to confirm it.

We further investigated the relationship between Nodal expression and clinicopathological characteristics in CRC, and found that Nodal was related with the advanced node stage and AJCC stage in CRC, which was consistent with the fact that Nodal promotes tumor growth in CRC [23]. Guinney et al recently proposed four subtypes (CMS classifications) for CRC based on the consolidation of six different large genomic subtyping studies [10]. Among the four CMSs, CMS4 (mesenchymal, 23%) is mainly manifested as MSS status, being a SCNA-high and CIMP-negative phenotype, occurring at advanced stages, having a poorer prognosis, and exhibiting TGF-β activation, EMT activation, stromal infiltration, angiogenesis and matrix remodeling [10, 12, 24]. However, the CMSs are dependent on special genetic test and difficult to be detected by routine pathological methods. Recent studies have shown that TSP was an independent prognostic factor for CRC and association with poor overall survival, and can be deemed as a good marker to determine CMS4 subtype of CRC patients in routine pathological examination [18]. As a member of TGF-β superfamily, Nodal was presumed to be overexpressed and be a diagnostic marker in CMS4 CRC. In the present study, we found that high Nodal expression was the only independent risk factor for high TSP in CRC. ROC curve confirmed the diagnostic value of Nodal for predicting high TSP. As high TSP is thought to be a valuable marker to determine CMS4, we might also speculate that Nodal could be another reliable diagnostic index in CMS4 CRC, especially when tumor specimens contain much necrosis or mucus tissue, which makes grading TSP very difficult. Moreover, high Nodal expression correlated with pMMR status, left colon cancer and advanced stages, which were in accordance with the features of CMS4 CRC and further confirmed the above hypothesis.

Several limitations exist in our research. Accurate diagnosis of CMS relies on specialized genetic testing, which is too costly and discommodious to be introduced into routine pathology, so we just took TSP as an alternative “gold standard” for diagnosing CMS4. Besides, we inferred that Nodal might participate in the procedure of malignant transformation of CRC based on the finding that Nodal only overexpressed in HGIN and CRC instead of other benign lesions and normal colon tissues, however, the potential mechanism research such as which signaling pathway does Nodal work through was missing, which need further investigation in the future.

Taken together, based on our research, Nodal might play a role in colorectal tumor progression and be used as a diagnostic marker to identify benign and malignant colorectal lesions under certain circumstances. Besides, Nodal might also be used as a marker for determining CMS4 subtype of CRC. We thus identified a potential driver of malignant transformation in colorectal lesions and a relatively simple and feasible method to determine the CMS4 subtype of CRC.

## Data Availability

The original contributions presented in the study are included in the article/Supplementary Material, further inquiries can be directed to the corresponding author.
